# Distribution and Genetic Characteristics of Seoul Virus in Different Organs of *Rattus norvegicus*

**DOI:** 10.3390/v17030412

**Published:** 2025-03-14

**Authors:** Yamei Wei, Xiaodong Shi, Yanan Cai, Zhanying Han, Yanbo Zhang, Yonggang Xu, Xu Han, Qi Li

**Affiliations:** Institute for Viral Disease Control and Prevention, Hebei Provincial Centre for Disease Control and Prevention, Shijiazhuang 050021, China; weiyamei2013@163.com (Y.W.); sxd15176139965@163.com (X.S.); yanan589@163.com (Y.C.); hzhyehf@163.com (Z.H.); zhyb1966@126.com (Y.Z.); walterxu04@sina.com (Y.X.)

**Keywords:** hantavirus, Seoul virus, *Rattus norvegicus*, pathogen distribution, genetic characteristic, viral tropism, multi-organ surveillance

## Abstract

To investigate the distribution of hantavirus (HV) in rodent organs, we selected eight counties across four regions in Hebei Province (southern, northern, eastern, and central) as study areas. Rodents were captured using night trapping methods, and organ samples were aseptically collected for HV detection via quantitative real-time PCR (qPCR) and gene sequencing. During the 2022–2023 spring and autumn seasons, 1386 rodents were trapped, including 73 *Rattus norvegicus* carrying Seoul virus (SEOV). The highest detection rate was observed in the liver (3.84%), followed by the kidneys (3.46%) and lungs (3.09%). Viral load analysis revealed higher SEOV RNA levels in the liver than in the lungs and kidneys. Antibody levels in *R. norvegicus* may influence the detection of viruses in organs. Phylogenetic analysis indicated that all sequences belonged to the S3 subtype, exhibiting regional aggregation and genetic stability. Our findings emphasize the necessity of multi-organ sampling for comprehensive HV surveillance and epidemic risk assessment.

## 1. Introduction

Hantaviruses (HVs), members of the class *Bunyaviricetes*, order *Elliovirales*, family *Hantaviridae*, genus *Orthohantavirus*, are enveloped, negative-sense RNA virus characterized by a tripartite genome (L, M, and S segments) [1,2,3]. These segments encode the RNA-dependent RNA polymerase (RdRp), envelope glycoproteins (Gn and Gc), and nucleocapsid protein (NP), respectively [4,5,6,7,8]. As the primary causative agent of hemorrhagic fever with renal syndrome (HFRS), HVs exhibit a case fatality rate of 1–15% [3,9,10], with China accounting for approximately 90% of global HFRS cases [5,11]. Seoul virus (SEOV), a type of HV, is endemic in Asia, Europe, and the Americas, with recent reports underscoring its expanding urban presence [12,13,14,15]. It poses a serious threat to public health and safety.

There are many species of HV hosts, such as rodents, shrews, and moles, etc., and HV shows strict host specificity, with certain rodent species serving as primary reservoirs. The number, species, and distribution of hosts determine not only the type of HV carried, the intensity of the epidemic, but also the virulence of the virus, clinical manifestations, and severity [9]. In China, *Apodemus agrarius* and *Rattus norvegicus* (*R. norvegicus*) are the principal hosts for Hantaan virus (HTNV) and SEOV, respectively [16].

Despite persistent HV infections in rodents, these hosts remain asymptomatic [17,18], yet they continuously shed infectious virions through saliva, urine, and feces [3,19]. Transmission to humans occurs via inhalation of aerosolized excreta or direct contact with mucosal membranes, underscoring the significant public health risks posed by HV [20,21,22,23].

Previous studies have predominantly focused on HV detection in lung and kidney tissues, as these organs are considered primary sites of viral replication [24,25]. However, recent evidence has identified HV RNA in the livers of rodents in China, Hungary, and the Netherlands [19,26,27]. It suggests a broader organ tropism than previously recognized. HV infection leads to multi-organ involvement through mechanisms including direct damage to vascular endothelial cells, dysregulated immune responses, and microcirculatory disturbances [28]. As critical components of the circulatory and immune systems, the heart and spleen are potential targets for viral invasion [29,30]. Systematic examination of these organs could elucidate the extent of viral tropism and tissue colonization. Nevertheless, current research into heart and spleen involvement remains sparse, with most studies limited to lung and excreted samples [31,32]. This knowledge gap hinders a comprehensive understanding of HV pathogenesis and its potential for multi-organ dissemination.

To address this limitation, we systematically analyzed the distribution and genetic characteristics of SEOV across multiple organs (lungs, liver, kidneys, heart, and spleen) in *R. norvegicus*. Our findings aim to refine HV surveillance strategies and elucidate viral persistence mechanisms in reservoir hosts, thereby informing targeted prevention measures against zoonotic transmission.

## 2. Materials and Methods

### 2.1. Ethics

The experimental protocol was approved by the Ethics Committee of the Hebei Provincial Center for Disease Prevention and Control. Rodents were humanely euthanized via cardiac puncture under isoflurane anesthesia, followed by aseptic collection of organ samples in accordance with institutional guidelines (IRB(P)2016-001).

### 2.2. Sample Collection

From 2022 to 2023, rodents were captured seasonally (spring and autumn) across eight counties spanning four geographical regions of Hebei Province (southern, northern, eastern, and central) using nocturnal trapping methods. The regions were chosen due to their ecological diversity, high rodent population density, and historical prevalence of HFRS cases in Hebei Province. Species identification was performed on-site, and organ samples (lungs, liver, kidneys, heart, and spleen) were aseptically collected. Specimens were recorded for collection date, location, habitat, and biological characteristics (species sex, and age), then transferred to numbered preservation tubes, flash-frozen in liquid nitrogen, and stored at −80 °C until further analysis. Blood samples were collected via cardiac puncture post-euthanasia, centrifuged at 3000× *g* for 10 min to separate serum, and stored at −80 °C for antibody testing.

### 2.3. Nucleic Acid Extraction

Organ tissues (50 mg) were homogenized in 1.5 mL EP tubes containing 1 mL saline and grinding beads using a high-throughput tissue homogenizer. The homogenate was centrifuged at 12,000× *g* for 10 min, and total RNA was extracted using the Berger Nucleic Acid Extraction and Purification Kit (Shanghai Berger Medical Technology Co., Ltd., Shanghai, China).

### 2.4. Quantitative Real-Time PCR (qPCR)

HV typing was performed using the AM1005 AgPath-ID™ One-Step RT-PCR Kit (Thermo Fisher Scientific, Waltham, MA, USA). RNA (5 μL) from each sample was added to reaction mixtures containing HV-specific primers and probes (Table 1). Cycling conditions included reverse transcription at 50 °C for 30 min, initial denaturation at 95 °C for 10 min, followed by 40 cycles of 95 °C for 15 s and 60 °C for 45 s. Primers and probes were designed based on conserved regions of HTNV and SEOV S segment [33]. The HTNV probe was labeled with a 5′-FAM fluorophore and 3′-BHQ1 quencher, while the SEOV probe utilized HEX/BHQ1.

### 2.5. Anti-Hantavirus Antibody Detection

Blood samples from *R. norvegicus* were screened for anti-hantavirus antibodies using the Anti-Hantavirus Total Antibody (IgG + IgM) Detection Kit (Xi’an Saitech Biotechnology Co., Ltd., Xi’an, China). This ELISA-based test detected anti-HV antibodies: blood loaded onto recombinant nuclear protein (rNP)-coated plates with controls, incubated (37 °C), washed, treated with HRP conjugate, developed with substrates (dark, 37 °C), and quantified by OD450 after stopping the reaction.

### 2.6. Whole Genome Sequencing

Positive organ samples (Ct ≤ 25) underwent whole genome amplification using the HFRS Hantavirus (Seoul Virus) Whole Genome Amplification Kit (Xi’an Saitech Biotechnology Co., Ltd., Xi’an, China). First-strand cDNA synthesis was performed with 16 μL RNA and 4 μL reaction mix under cycling conditions: 25 °C (10 min), 50 °C (10 min), and 85 °C (5 min). Second-round amplification used 5 μL of the first-round product in a 45 μL reaction mixture, with cycling parameters: 95 °C (4 min), 5 cycles of 94 °C (30 s), 50 °C (1 min), 72 °C (2 min), followed by 35 cycles of 94 °C (30 s), 55 °C (1 min), 72 °C (2 min), and a final extension at 72 °C (10 min). Sequencing was conducted on a NextSeq 2000 platform (Illumina, San Diego, CA, USA).

### 2.7. Phylogenetic Analysis

Sequences were aligned with GenBank references using Clustal W in MEGA 11. Maximum likelihood trees were constructed using the best-fit substitution model, with bootstrap support assessed via 1000 replicates. Reference sequences were selected based on geographic relevance (Asia, Europe, Americas), host species (*R. norvegicus*), and genomic completeness (full-length L, M, S segments).

### 2.8. Statistical Analysis

Differences in SEOV detection rates across organs were analyzed using chi-squared tests, with significance defined as *p* < 0.05.

## 3. Results

### 3.1. Detection of HV in Rodents

A total of 1386 rodents were captured across the study area during the spring and autumn seasons of 2022–2023, including 1076 *R. norvegicus*, 291 *Mus musculus*, 17 *Rattus tanezumi*, and 2 *Cricetulus triton*. Organ samples (lungs, livers, kidneys, hearts, and spleens) were collected, yielding 1386 lung, 1068 liver, 1231 kidney, 693 heart, and 474 spleen specimens. Among these, 73 rodents tested positive for HV, with positive samples distributed across organs as follows: lungs (38), livers (41), kidneys (38), hearts (4), and spleens (19). Detection rates for each organ were calculated as 2.74% (lungs), 3.84% (livers), 3.09% (kidneys), 0.58% (hearts), and 4.01% (spleens). Notably, all HV-positive rodents were identified as *R. norvegicus* carrying SEOV. Among 73 SEOV-positive *R. norvegicus* samples, 65.8% were male (*n* = 48) and 34.2% female (*n* = 25). A chi-squared test revealed no statistically significant difference in sex distribution between this group and the overall captured population (58.2% male; χ^2^ = 1.829, *p* = 0.109). Age distribution showed a clear predominance of adult individuals (*n* = 69, 92.2%) compared to young specimens (*n* = 4, 5.8%). Due to the limited number of young samples (*n* = 4), formal statistical comparisons were not conducted.

### 3.2. Detection of SEOV in Different Organ Samples

**Figure 1:**
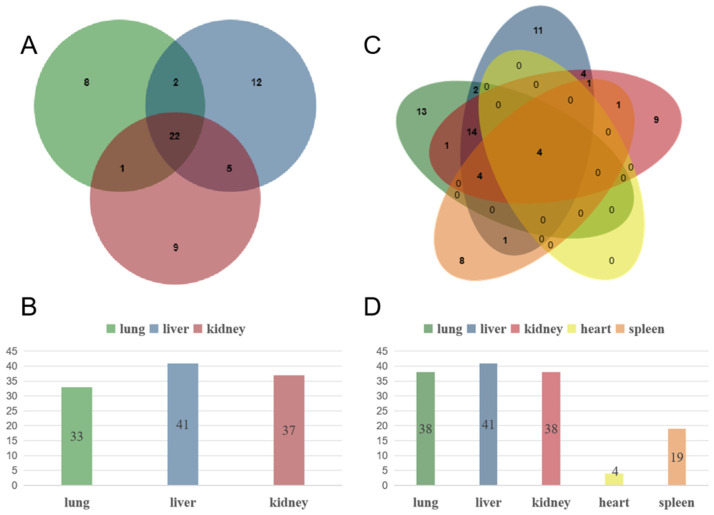
Detection of SEOV in different organs. (**A**) Absolute counts of positive samples for lung, liver, and kidney; (**B**) detection rates (positive samples/total samples tested) for lung, liver, and kidney; detection of SEOV in five organs (lungs, liver, kidneys, heart, spleen) of *Rattus norvegicus*; (**C**) absolute counts of positive samples for lung, liver, kidney, heart, and spleen; (**D**) detection rates (positive samples/total samples tested) for lung, liver, kidney, heart, and spleen.

**Figure 2:**
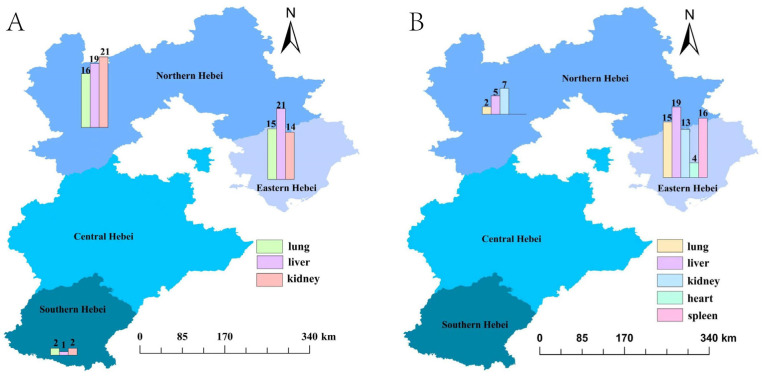
Regional distribution of lung, liver, and kidney samples (**A**) and lung, liver, kidney, heart, and spleen samples (**B**) in SEOV positive *R. norvegicus*. Map source: The Chinese Academy of Surveying and Mapping.

Among 1068 *R. norvegicus* with lung, liver, and kidney samples, 59 *R. norvegicus* tested positive for SEOV via qPCR. These positive cases comprised 111 organ samples: 33 lungs, 41 livers, and 37 kidneys (Figure 1A,B). Twenty-two *R. norvegicus* exhibited SEOV positivity in all three organs. Chi-squared analysis revealed no statistically significant differences in detection rates among the liver, lung, and kidney (χ^2^ = 0.896, *p* = 0.639). Geographically, positive samples were predominantly distributed in the northern and eastern regions of Hebei Province (Figure 2A).

For the subset of 474 *R. norvegicus* with samples from all five organs (lungs, livers, kidneys, hearts, and spleens), 44 *R. norvegicus* tested positive for SEOV, yielding 84 positive organ samples: 17 lungs, 24 livers, 20 kidneys, 4 hearts, and 19 spleens (Figure 1C,D). Four individuals showed SEOV positivity in all five organs. Significant differences in detection rates were observed among organs (χ^2^ = 14.243, *p* = 0.007), with the heart exhibiting the lowest detection rate. Regional analysis indicated that heart samples had the lowest positivity in eastern Hebei (χ^2^ = 10.072, *p* = 0.039), while heart and spleen samples showed reduced detection rates in northern Hebei (χ^2^ = 14.586, *p* = 0.006) (Table 2 and Table 3). Positive samples were primarily clustered in the eastern and northern regions (Figure 2).

Cohen’s kappa analysis of SEOV detection in lung, liver, and kidney samples from *R. norvegicus* revealed poor agreement across organs: lungs vs. livers (κ = 0.155, *p* = 0.216), lungs vs. kidneys (κ = 0.251, *p* = 0.047), and livers vs. kidneys (κ = 0.173, *p* = 0.178). These results indicate that the presence of viral RNA in one organ does not reliably predict infection in others.

### 3.3. SEOV RNA Loads in Different Organs

**Figure 3:**
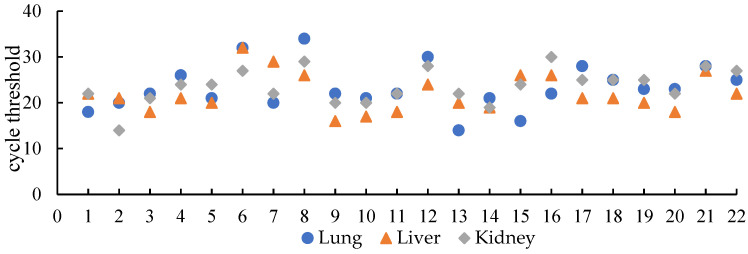
Measurement of SEOV RNA loads in different organs of *R. norvegicus*. (The lower the Ct value, the higher the viral load. The Ct values for all samples represent the average of three repeated tests).

Repeated qPCR analysis of lungs, livers, and kidneys from 22 SEOV-positive *R. norvegicus* revealed viral load variations across organs. The liver displayed the highest SEOV RNA levels (lowest Ct values) in 15 individuals (68.18%), followed by the lungs (22.72%) (Figure 3).

### 3.4. Anti-Hantavirus Antibody in the Blood of R. norvegicus

Anti-hantavirus total antibodies (IgG + IgM) were detected in 32 of 254 *R. norvegicus* blood samples (positivity rate: 12.60%). Among these, 18 rodents tested positive for SEOV in at least one organ (7.09% positivity). Notably, 24 antibody-positive individuals showed negative qPCR results in organs, while 10 antibody-negative rodents exhibited organ positivity.

### 3.5. Phylogenetic Analysis

**Figure 4:**
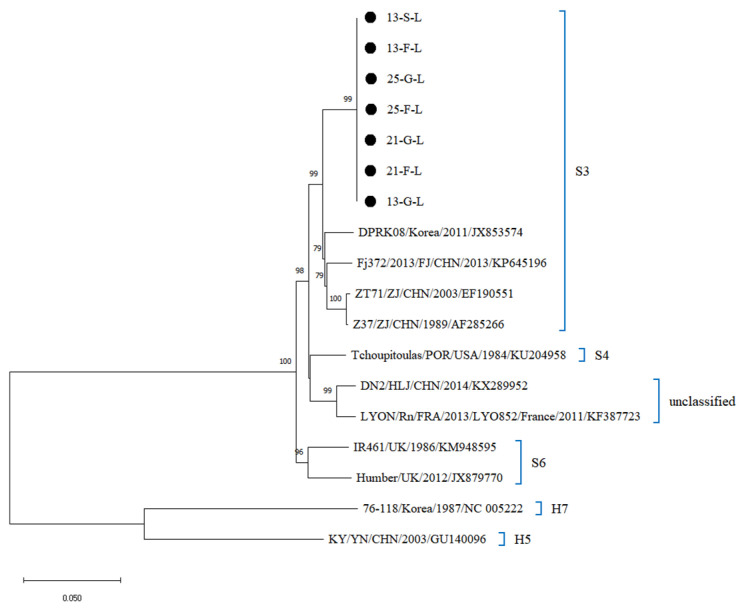
Phylogenetic tree constructed using nucleotide sequences of the SEOV L segment obtained from *R. norvegicus*.

**Figure 5:**
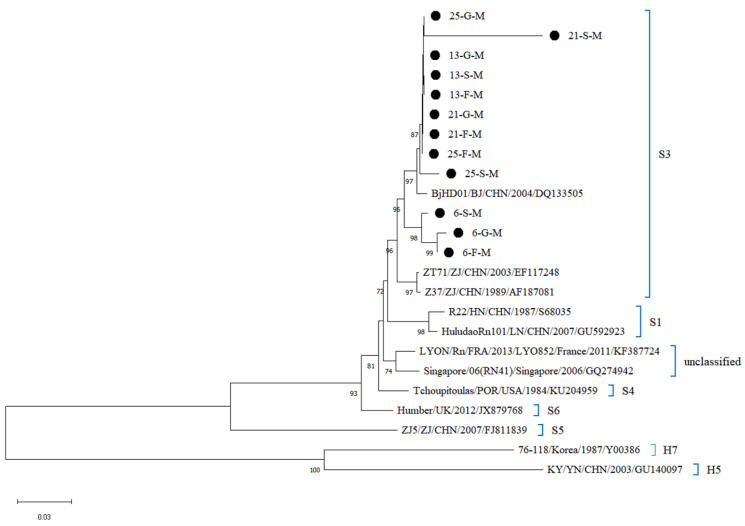
Phylogenetic tree constructed using nucleotide sequences of the SEOV M segment obtained from *R. norvegicus*.

**Figure 6:**
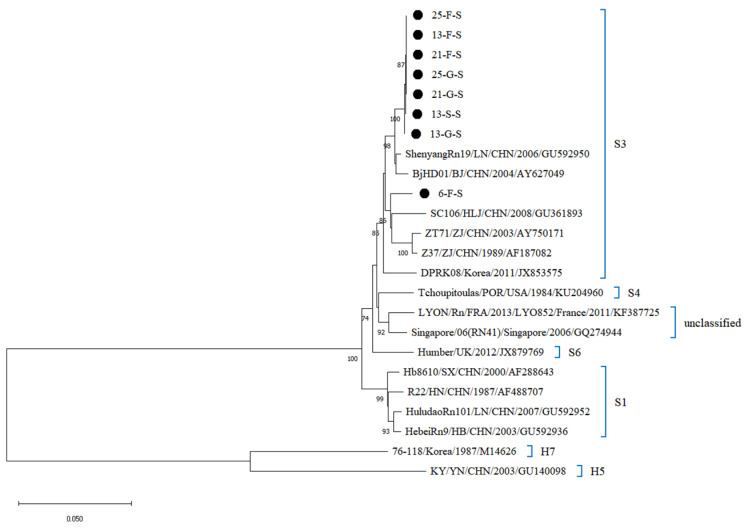
Phylogenetic tree constructed using nucleotide sequences of the SEOV S segment obtained from *R. norvegicus*.

Whole-genome sequencing of 27 SEOV-positive organ samples (Ct ≤ 25) from four *R. norvegicus* yielded 27 sequences, including 7 L-segment, 12 M-segment, and 8 S-segment sequences. The four *R. norvegicus* were samples 13, 21, and 25 from northern Hebei and sample 6 from southern Hebei. Phylogenetic reconstruction classified all sequences into the S3 subtype, clustering closely with strains from South Korea (DPRK08), Beijing (BjHD01), Fujian (Fj372/2013), Zhejiang (ZT71, Z37), and Liaoning (ShenyangRn19) (Figure 4, Figure 5 and Figure 6). Sequences from southern and northern Hebei occupied distinct evolutionary branches within the S3 subtype, reflecting regional genetic diversity. Comparative analysis of L segment sequences from different organs (lungs, liver, kidneys) of the same host showed negligible genetic divergence (nucleotide homology: 99.9–100%). In contrast, inter-host comparisons (e.g., northern vs. southern Hebei samples) exhibited M and S segment homology of 96.3–97.1%, and 95.7–97.8%, suggesting geographic-driven microevolution. Furthermore, the L gene sequences from Hebei SEOV strains demonstrated lower genetic diversity (average divergence: 0.1%) compared to Zhejiang (ZT71) and Liaoning (ShenyangRn19) strains, supporting regional transmission stability.

## 4. Discussion

Recent studies on HV detection in rodent organs beyond the lungs remain limited. Our study systematically investigated HV distribution across multiple organs of *R. norvegicus*, providing novel insights into viral tropism and persistence.

All 73 HV-positive rodents identified in this study were *R. norvegicus* carrying SEOV. *R. norvegicus* are known to be highly adaptable and migratory, allowing them to spread globally through human activities, thus facilitating the global transmission of SEOV [34,35]. Although no significant association between SEOV infection and rodent sex was found in this study, the higher proportion of males (65.75%) may be related to a wider range of individual males and a higher risk of exposure. However, the low infection rate of young individuals may reflect the limited mother-to-child transmission or the immune protection mechanism of the young, which needs further verification.

Previous studies have shown that the lung is the preferred organ for HV detection, with large amounts of HV RNA and antigens detectable in the lungs, which play a critical role in maintaining HV during persistent infection [36]. However, emerging evidence, including our findings, highlights broader organ tropism [26,37,38]. We detected SEOV in the lungs, liver, kidneys, heart, and spleen, with the highest detection rate in the liver (3.84%), followed by the lungs (3.09%) and kidneys (3.46%). Our detection of SEOV in multiple organs aligns with findings by Jiang [39], who identified HV RNA in the liver and spleen of *R. norvegicus*. Notably, viral load analysis revealed that 68.18% of *Rattus norvegicus* exhibited the highest SEOV RNA levels in the liver compared to the lungs and kidneys. These results suggest the liver serves as a major reservoir for HV replication, challenging the traditional focus on the lung and kidney. However, the concentration of a specific virus differs depending on the virus, the host and the organ tested [40]. Importantly, not all organs within an individual rodent tested positive simultaneously. The lack of agreement in SEOV detection across organs suggests that viral replication is compartmentalized, potentially due to tissue-specific barriers or immune modulation. This compartmentalization may facilitate viral persistence by evading systemic immune responses. The results underscore the necessity of multi-organ sampling to comprehensively assess HV infection status and transmission risks.

The overall antibody detection rate in this study (12.60%) was higher than the qPCR detection rate (7.09%). Discordant results were observed: some rodents were qPCR-positive but antibody-negative, potentially indicating early infection prior to seroconversion, consistent with previous studies by Easterbrook, Klein, and Németh [19,41,42,43]. Conversely, antibody-positive but qPCR-negative cases may reflect persistent infections with immune-mediated suppression of viral replication [17]. These findings align with the hypothesis that HV employs mechanisms to evade host immunity, enabling long-term persistence in reservoir hosts without overt pathology. Additionally, they suggest that antibody levels may influence the organ viral detection, influencing surveillance strategies. It underscores the need to integrate serological and molecular data in HV surveillance protocols.

Phylogenetic analysis of SEOV sequences from lung, liver, and kidney samples (Ct ≤ 25) revealed that all 27 sequences clustered within the S3 subtype, closely related to strains from South Korea (DPRK08), Beijing (BjHD01), and other Chinese provinces (Fujian, Zhejiang, Liaoning). This close relationship may be related to the frequent transmission of HV and the close connections between regions due to the development of the transport industry. These regions, including Hebei Province, are part of a mixed infection zone dominated by SEOV and HFRS, reflecting the geographic clustering and genetic stability of HV, which is consistent with previous domestic and international studies [34,44,45]. On the other hand, the clustering of sequences on distinct evolutionary branches within the S3 subtype highlights genetic diversity, likely driven by frequent viral transmission and human-mediated rodent dispersal.

Although we have carried out some research, this study still has some limitations, mainly the small sample size as it only included data from Hebei Province and used *R. norvegicus* as the host species. This limits the generalizability of the findings. To address this limitation, future research should increase the sample size by including samples from additional provinces and other rodent species. This will provide a more comprehensive understanding of the geographic distribution and host diversity of the virus. In addition, long-term monitoring and the use of a variety of methodologies will strengthen the results of the study and increase its overall accuracy and depth.

## 5. Conclusions

Our study demonstrated that SEOV exhibits differential prevalence across five organs (lungs, liver, kidneys, heart, and spleen) in *R. norvegicus*. Among these, the liver exhibited the highest detection rate and viral load (based on Ct values). These findings highlight the liver as a critical target organ for HV replication, challenging the conventional emphasis on the lung and kidney. Antibody levels in rodents may influence the detection of viruses in organs, suggesting a potential immune-mediated suppression of viral replication during persistent infections. Clustering of the S3 subtype reveals a geographic transmission linkage, with both stability and diversity observed. Multi-organ sampling is critical to avoid underestimating HV prevalence and tropism, guiding targeted strategies to reduce zoonotic risks.

## Figures and Tables

**Table 1:** Primer and probe sequences of HV.

Category	Primer/Probe	Sequence (5′→3′)
HTNV	HTNV-F	GCTTCTTCCAGATACAGCAGCAG
	HTNV-R	GCCTTTGACTCCTTTGTCTCCAT
	HTNV-P	CCTGCAACAAACAGGGAYTACTTACGGCA
SEOV	SEOV-F	GATGAACTGAAGCGCCAACTT
	SEOV-R	CCCTGTAGGATCCCGGTCTT
	SEOV-P	CCGACAGGATTGCAGCAGGGAAGAA

**Table 2:** Detection of lung, liver, and kidney samples in different regions.

Region	Number of Detections	Number of Positives (Detection Rate)	Number of Positives (Detection Rate)		
			Lung	Liver	Kidney
Eastern Hebei	387	32 (8.27%)	15 (3.88%)	21 (5.43%)	14 (3.62%)
Central Hebei	312	0	0	0	0
Southern Hebei	134	2 (1.49%)	2 (1.49%)	1 (0.75%)	2 (1.49%)
Northern Hebei	235	25 (10.64%)	16 (6.81%)	19 (8.09%)	21 (8.94%)
Total	1068	59 (5.52%)	33 (3.09%)	41 (3.84%)	37 (3.46%)

**Table 3:** Detection of five kinds of organs in different regions.

Region	Number of Detections	Number of Positives (Detection Rate)	Number of Positives (Detection Rate)				
			Lung	Liver	Kidney	Heart	Spleen
Eastern Hebei	314	34 (10.83%)	15 (4.78%)	19 (6.05%)	13 (4.14%)	4 (1.27%)	16 (5.10%)
Central Hebei	104	3 (2.89%)	0	0	0	0	3 (2.88%)
Northern Hebei	56	7 (12.50%)	2 (3.57%)	5 (8.93%)	7 (12.5%)	0	0
Total	474	44 (9.28%)	17 (3.59%)	24 (5.06%)	20 (4.22%)	4 (0.84%)	19 (4.01%)

## Data Availability

All the sequences have been submitted to the Genbank.
